# SF3A1 and pancreatic cancer: new evidence for the association of the spliceosome and cancer

**DOI:** 10.18632/oncotarget.5647

**Published:** 2015-10-15

**Authors:** Jing Tian, Yaping Liu, Beibei Zhu, Yao Tian, Rong Zhong, Wei Chen, Xinghua Lu, Li Zou, Na Shen, Jiaming Qian, Hui Li, Xiaoping Miao, Li Wang

**Affiliations:** ^1^ Department of Epidemiology and Biostatistics, Institute of Basic Medical Sciences Chinese Academy of Medical Sciences, School of Basic Medicine Peking Union Medical College, Beijing, China; ^2^ State Key Laboratory of Environment Health (Incubation), MOE (Ministry of Education) Key Laboratory of Environment & Health, Ministry of Environmental Protection Key Laboratory of Environment and Health (Wuhan), and Department of Epidemiology and Biostatistics, School of Public Health, Tongji Medical College, Huazhong University of Science and Technology, Wuhan, China; ^3^ Division of Gastroenterology, Peking Union Medical College Hospital, Chinese Academy of Medical Sciences, Peking Union Medical College, Beijing, China

**Keywords:** pancreatic cancer, RNA splicing, genetic variants, smoking, drinking

## Abstract

A two-stage case-control study was conducted to examine the association between six candidate U2-depedent spliceosome genes (SRSF1, SRSF2, SF3A1, SF3B1, SF1 and PRPF40B) and pancreatic cancer (PC). Subjects with one or two T alleles at rs2074733 in SF3A1 had a lower risk of PC compared to those with two C alleles in combined two populations (OR: 0.59, 95% confidence interval: 0.48–0.73, False discovery rate (FDR)-*P* = 1.5E-05). Moreover, the presence of the higher-risk genotype at rs2074733 plus smoking or drinking had synergic effects on PC risk. These findings illustrate that RNA splicing-related genes appear to be associated with the occurrence of PC, and show synergic interactions with smoking and drinking in the additive model. In the future, our novel findings should be further confirmed by functional studies and independent large-scale population studies.

## INTRODUCTION

Pancreatic cancer (PC), which is a highly malignant tumor associated with poor prognosis [1], is the fourth leading cause of cancer death in the United States, with an estimated 38,460 deaths in 2013 [2]. Also, the mortality rate in China have been risen gradually since 1991 [3]. PC is caused by the interaction of environmental and genetic factors. Although many environmental factors (e.g., cigarette smoking, alcohol drinking and dietary factors) have been associated with the development of PC, smoking is the only confirmed factor [1, 4]. With respect to the germline variation of PC, multiple genes including BRCA2, PALB2, ATM, the ABO loci, NR5A2, CLPTM1L-TERT, BACH1, DAB2, and FAM19A5, have been shown to be associated with the progression of PC [1, 5–9]. However, although many genetic factors have been identified, their PC-related interactions with environmental factors have not yet been fully elucidated.

As the field of cancer genomics continues to expand, scores of new cancer genes have been revealed, including genes involved in RNA splicing [10]. RNA splicing is an essential function in almost all eukaryotic organisms. During this process, introns are removed from pre-messenger RNAs; splicing factors recognize specific-sequences in the pre-RNA; and the spliceosome connects the exons. There are two types of spliceosomes, the major U2-dependent spliceosomes and the minor U12-dependent spliceosomes, which recognize the major U2- and minor U12-dependent intron groups, respectively [11]. During U2-dependent intron recognition, a number of factors play prominent roles, including: the U1 small nuclear ribonucleoproteins (U1 snRNPs) responsible for recognizing 5′-splice site (5′SS); splicing factor1 (SF1), which binds the branch-site A residue; the U2 small nuclear ribonucleoprotein auxiliary factor 35/65 (U2AF35/65) heterodimer responsible for recognizing the 3′-splice site (3′SS) by binding the AG dinucleotide and the downstream polypyrimidine tract of the branch site; and the serine-arginine (SR) rich family of RNA-binding proteins (e.g., SRSF1 and SRSF2), which can bind to splicing enhancers through the arginine -serine rich domain and recruit the U1 snRNP and U2AF to the 5′ or 3′SS. SF3B1, splicing factor 3b, subunit 1, which encodes the SF3b1 protein, helps the U2 snRNP bind to the 3′SS with SF3a1 (Supplementary Figure S1).

Recent studies have shown that some spliceosome genes involved in the early steps of U2-dependent splice site recognition are commonly mutated in hematologic malignancies and solid cancers [12–18]. For example, exome-sequencing studies found that SF3B1 was mutated in 10–15% of patients with chronic lymphocytic leukemia, while other spliceosome genes (e.g. SRSF1, SRSF7 and U2AF65) were mutated at lower (but still detectable) frequencies in chronic lymphocytic leukemia patients [12, 13]. In myelodysplastic syndrome, spliceosome genes were reported to be mutated in 45–85% of patients; mutations were commonly found in SF3B1, SRSF2 and U2AF35, and also found (albeit at lower frequencies) in SF3A1, PRPF40B, U2AF65 and SF1 [14]. Finally, the U2-dependent spliceosome proteins, SF3B1, U2AF35, U2AF65, and PRRF40B, are significantly mutated in solid tumors of lung adenocarcinomas [15], while mutations in SF3B1 have been associated with breast cancer, uveal melanoma, and PC [16–18].

Although genetic variants of the spliceosome complex are generally believed to play a causative role in cancer, relatively few association studies have been performed on solid tumors, especially PC. We have already verified the association between genetic polymorphism of U2AF35/U2AF65 with PC and revealed the interaction of U2AF65 and smoking may increase the risk of PC previously [19]. Subjects with C allele in rs310445 of U2AF65 gene had a 1.31-fold risk to be associated with pancreatic cancer compared to those with TT genotype (*p* = 0.010). A synergic effect of smoking and C allele of rs310445 was also observed, with synergic index (SI) of 2.08 (95% confidence interval (CI): 1.37–2.78) [19]. Here, we selected other six U2-dependent spliceosome genes previously shown to have cancer-associated mutations (SRSF1, SRSF2, SF3A1, SF3B1, SF1 and PRPF40B), and screened 17 putative tag single nucleotide polymorphisms (tagSNPs) in these genes. We used a two-stage case-control study, in which two independent Chinese populations were taken as the screening and validation populations, and used to explore the genetic effect of U2-dependent spliceosomes on PC susceptibility. We also examined potential gene-environment interactions between the identified variant, smoking status, drinking status, and PC risk.

## RESULTS

### Subject characteristics

Five samples (2 cases and 3 controls) with call rates less than 95% were excluded. 298 PC cases and 525 controls were finally included in the screening stage, and 413 PC cases and 557 controls were included in the validation stage. The characteristics of the subjects are summarized in Table 1. There was no significant difference in the age or gender distribution between PC patients and controls in either population. The proportions of smokers and drinkers among the cases were higher than those among the controls. However, only smoking was significantly associated with the risk of PC in both populations. The odds ratios (ORs) for smoking in PC patients of the screening and validation populations were 1.82 (95% CI: 1.34–2.49) and 1.36(95% CI: 1.04–1.76), respectively.

**Table 1:** Characteristics of subjects in this two-stage case-control study

	Screening Stage			Validation Stage		
	Controls (*N* = 525)	Cases (*N* = 298)	*P*	Controls (*N* = 557)	Cases (*N* = 413)	*P*
Age (mean ± SD) years	58.9 ± 12.9	60.3 ± 12.8	0.132	59.0 ± 13.3	58.6 ± 13.1	0.630
Gender						
Male(%)	260(49.5)	164(55.0)	0.129	341(61.2)	254(61.5)	0.929
Female(%)	265(50.5)	134(45.0)		216(38.8)	159(38.5)	
Smoking status						
Never(%)	403(76.8)	192(64.4)	**< 0.001**	365(65.5)	241(58.4)	**0.023**
Ever(%)	122(23.2)	106(35.6)		192(34.5)	172(41.7)	
Drinking status						
Never(%)	412(78.5)	219(73.5)	0.104	376(67.5)	275(66.6)	0.763
Ever(%)	113(21.5)	79(26.5)		181(32.5)	138(33.4)	

### Genotyping and assessing the associations between SNPs and PC

In the screening population, we detected the genotyping of 17 tagSNPs in six candidate genes. Three SNPs (rs11231868 in SF1, rs5749066 and rs5749068 in SF3A1) deviated significantly from Hardy-Weinberg Equilibrium (HWE) in the control group [False discovery rate (FDR)-P < 0.05], and were thus excluded from the analysis (Supplementary Table S1). Therefore, 14 SNPs were included in our analysis. Among them, six SNPs in three genes showed potential associations with PC: rs4073998 and rs8626 in PRPF40B; rs2074733, rs5994293 and rs9608886 in SF3A1; and rs8819 in SRSF1. These SNPs were significantly different between the patient and control groups after we adjusted for age, gender (Supplementary Table S2). The same trend was still existed after adjusted for age, gender, smoking and drinking. The P values after FDR correction were 0.014, 5.5E-05, 0.014, 0.023, 0.014 and 0.001, respectively. The ORs and 95%CIs are summarized in Table 2. Further analysis showed evidence for strong linkage disequilibrium between rs4073998 and rs8626 (D' = 0.964, *r* ^2^ = 0.717). Therefore, five SNPs (rs4073998 in PRPF40B; rs2074733, rs5994293 and rs9608886 in SF3A1 and rs8819 in SRSF1) were selected for further validation.

**Table 2:** Effects of 14 tag SNPs from eight RNA splicing-related genes on PC risk in the screening population

Gene	SNP	Genotypes^a^	Controls (*N* = 525) (%)	Cases (*N* = 298) (%)	OR (95%CI)^b^	*P*	FDR-*P*^c^
*PRPF40B*	rs4073998	(AA+AG)/GG	65(12.4)/460(87.6)	59(19.8)/239(80.2)	1.80(1.22–2.67)	0.003	0.014*
	rs8626	(GG+AG)/AA	71(13.5)/454(86.5)	78(26.2)/220(73.8)	2.37(1.64–3.41)	3.9E-06	5.5E-05*
*SF1*	rs474707	(TT+CT)/CC	320(61.0)/205(39.0)	178(59.7)/120(40.3)	0.96(0.71–1.28)	0.757	0.815
*SF3A1*	rs12484880	(GG+TG)/TT	76(14.5)/449(85.5)	47(15.8)/251(84.2)	1.11(0.74–1.66)	0.610	0.712
	rs2074733	(TT+CT)/CC	366(69.7)/159(30.3)	179(60.1)/119(39.9)	0.65(0.48–0.88)	0.005	0.014*
	rs5753081	CT/CC	29(5.5)/496(94.5)	15(5.0)/283(95.0)	0.97(0.51–1.85)	0.928	0.928
	rs5994293	GG/GT/TT	56(10.7)/200(38.1)/269(51.2)	19(6.4)/102(34.2)/177(59.4)	0.74(0.59–0.93)	0.01	0.023*
	rs7288947	(TT+CT)/CC	166(31.6)/359(68.4)	76(25.5)/222(74.5)	0.75(0.54–1.03)	0.075	0.131
	rs8141656	(CC+CT)/TT	261(49.7)/264(50.3)	164(55.0)/134(45.0)	1.25(0.94–1.67)	0.130	0.202
	rs9608886	(GG+TG)/TT	113(21.5)/412(78.5)	40(13.4)/258(86.6)	0.55(0.37–0.82)	0.004	0.014*
*SF3B1*	rs11683572	(GG+CG)/CC	222(42.3)/303(57.7)	108(36.2)/190(63.8)	0.74(0.55–1.00)	0.047	0.094
*SRSF1*	rs2233911	(GG+AG)/AA	170(32.4)/355(67.6)	114(38.3)/184(61.7)	1.25(0.92–1.68)	0.151	0.211
	rs8819	CT/CC	21(4.0)/504(96.0)	32(10.7)/266(89.3)	3.04(1.69–5.48)	2.1E-04	0.001*
*SRSF2*	rs237059	CT/CC	6(1.1)/519(98.9)	1(0.3)/297(99.7)	0.26(0.03–2.18)	0.212	0.270

a The last genotype was used as the reference for OR estimation.

b Adjusted by gender, age, smoking and drinking in the unconditional logistic regression.

c Each *P* value was modified by FDR correction for multiple comparisons (the number of comparisons = 14).

* Significant difference after FDR correction

Genotyping of these five SNPs was performed in the larger validation population of 413 PC cases and 557 controls. All of the tested SNPs conformed to HWE in the control group. Interestingly, only rs2074733 was significantly different between PC cases and controls after we adjusted for age, gender, smoking and drinking. Compared with the CC genotype, subjects with the TT or CT genotypes had a lower risk of PC [OR 0.54 (95%CI: 0.41–0.73); FDR-P = 3.0E-04] (Table 3).

**Table 3:** Effects of five candidate tagSNPs on PC risk in the validation population

Gene	SNP	Genotypes^a^	Controls (*N* = 557) (%)	Cases (*N* = 413) (%)	OR (95%CI)^b^	*P*	FDR-*P*^c^
PRPF40B	rs4073998	(AA+AG)/GG	67(12.0)/490(88.0)	54(13.1)/359(86.9)	1.12(0.76–1.65)	0.563	0.669
SF3A1	rs2074733	(TT+CT)/CC	434(77.9)/123(22.1)	272(65.9)/141(34.1)	0.54(0.41–0.73)	3.2E-05	3.0E-04*
	rs5994293	GG/GT/TT	37(6.6)/217(39.0)/303(54.4)	18(4.4)/154((37.3)/241(58.3)	0.85(0.69–1.06)	0.145	0.221
	rs9608886	GT/TT	18(3.2)/539(96.8)	21(5.1)/392(94.9)	1.64(0.86–3.14)	0.132	0.221
SRSF1	rs8819	(CT+TT)/CC	21(3.8)/536(96.2)	10(2.4)/403(97.6)	0.62(0.29–1.33)	0.221	0.280

a The last genotype was used as the reference for OR calculations.

b Adjusted by gender, age, smoking and drinking in the unconditional logistic regression.

c Each *P* value was modified by FDR correction for multiple comparisons (the number of comparisons = 19)

* Significant difference after FDR correction.

We further analyzed the effect of rs2074733 in the combined populations of the two stages, and the resultant P value for the Breslow-Day homogeneity test was 0.396. In agreement with the abovementioned results, rs2074733 was significantly associated with PC risk in the combined population. Subjects with one or two T alleles had a lower risk for PC compared to those with CC genotype [OR 0.59(95%CI: 0.48–0.73), FDR-P = 1.5E-05].

### Gene-environment interactions with respect to PC risk

Table 4 shows the results of our multiplicative and additive interaction analyses between rs2074733 and smoking or drinking in the combined group. Smoking had a synergic additive interaction with the CC genotype of rs2074733. Compared to nonsmokers with one or two T alleles, there were higher risks of PC among smokers with one or two T alleles, nonsmokers with the CC genotype, and smokers with the CC genotype. The ORs were 1.53 (95%CI: 1.12–2.08), 1.45 (95%CI: 1.12–1.88) and 3.05 (95%CI: 2.07–4.50), respectively. The SI, the attributable proportion due to interaction (AP) and the relative excess risk due to interaction (RERI) were 2.43 (95%CI: 1.85–3.01), 1.38 (95%CI: 0.37–2.40), and 0.41 (95%CI: 0.22–0.61), respectively. Moreover, we observed an positive additive interaction between drinking and rs2074733 in the combined population too. Compared to non-drinkers with one or two T alleles, the ORs for drinkers with one or two T alleles, non-drinkers with the CC genotype, and drinkers with the CC genotype were 0.84 (95%CI: 0.61–1.14), 1.50 (95%CI: 1.17–1.92) and 1.68 (95%CI: 1.16–2.43), respectively. The SI was 2.44 (95%CI: 1.06–3.81).

**Table 4:** Interactions between smoking, drinking and rs2074733 in the occurrence of PC in the combined group

		Controls (%)	Cases (%)	OR (95%CI)^a^	*P_LLRT_*	SI (95%CI)	AP (95%CI)	RERI (95%CI)
**Smoking**								
Never	TT+CT	561(51.9)	285(40.1)	Ref	0.060	2.43(1.85–3.01)*	0.41(0.22–0.61)*	1.38(0.37–2.40)*
Ever	TT+CT	239(22.1)	166(23.4)	1.53(1.12–2.08)				
Never	CC	207(19.1)	148(20.8)	1.45(1.12–1.88)				
Ever	CC	75(6.9)	112(15.8)	3.05(2.07–4.50)				
**Drinking**								
Never	TT+CT	583(53.9)	328(46.1)	Ref	0.106	2.44(1.06–3.81)*	0.26(−0.02−0.53)	0.45(−0.14−1.03)
Ever	TT+CT	217(20.1)	123(17.3)	0.84(0.61–1.14)				
Never	CC	205(19.0)	166(23.4)	1.50(1.17–1.92)				
Ever	CC	77(7.1)	94(13.2)	1.68(1.16–2.43)				

a Adjusted by gender, age, stage and drinking or smoking.

* Statistically significant.

## DISCUSSION

This two-stage case-control study in two independent Chinese populations investigated whether the potential functional SNPs of six U2-dependent spliceosome genes could be associated with PC. A reproducible association between rs2074733 of SF3A1 and PC risk in both populations was identified. And potential synergic additive interactions between CC genotype and smoking/drinking status were found. This is the first study to show a SNP of SF3A1 and its interaction with smoking/drinking and the risk of PC in central and northern Chinese populations.

SF3A1 encodes subunit 1 of the splicing factor 3a heterotrimer, which can facilitate the binding of the U2 snRNP to the 3′SS with SF3b1. Studies have shown that the SF3a heterotrimer is necessary for the *in vitro* conversion of the 15S U2 snRNP into the active 17S particle, which performs pre-mRNA splicing and the knockdown of single SF3 subunits blocks splicing [20]. Population studies have revealed that SF3A1 expression may be up-regulated in head and neck cancers, rectal carcinomas, and human non-small and small-cell lung cancers [21–23]. The aberrant expression of splicing factors, including SF3A1, are known to modify splice site selection [24], thereby influence the splicing of oncogenes and tumor suppressors, and induce the production of mRNA isoforms. This could contribute directly or indirectly to the development, progression and therapeutic response of cancers [25]. For example, the abnormal expression of SR family proteins (e.g., SRSF1 and SRSF3) in various human tumors was found to affect the alternative splicing of cancer-related genes, such as RON, BIN1, MNK2, S6K1, KLF6, FoxM1 and HIPK2, thereby creating protein isoforms that could contribute to the proliferation, avoidance of apoptosis, cell cycle modulation, or signal transduction of tumor cells [26–30]. Through our bioinformatic analysis, three SNPs rs5753071, rs10376 and rs10427610 were in complete linkage disequilibrium with rs2074733. They were reported to locate at the site of transcription factor binding, histone modification and open chromatin. And evidence suggested they acted as expression quantitative trait loci for the SF3A1 gene [31]. Notably, rs2074733 is located at 22q12.2, which is reportedly associated with the occurrence of lung cancer in Han Chinese [32]. Thus, genetic variants rs2074733 in SF3A1 may be involved in other cancer types beyond PC. Future studies are warranted to determine whether changes in SF3A1 can alter the splicing of specific oncogenes or tumor suppressors to promote PC.

We observed synergistic effects between smoking/drinking and rs2074733. These findings seem biologically plausible. First, smoking is the only confirmed environmental risk factor for PC. Although the underlying mechanisms are poorly understood, a higher frequency of splicing variants for the MDM2 oncogene has been observed in tumors induced in smokers than those in non-smokers [33]. Its over expression reportedly contributes to pancreatic neoplastic transformation [34], and its splicing variants may promote p53-independent cell growth, inhibit apoptosis and contribute to tumorigenesis [35]. Furthermore, an *in vitro* study showed that the activated carcinogenic metabolites of polycyclic aromatic hydrocarbons can induce alternative splicing of MDM2 [33]. In the context of drinking, alcohol consumption has been shown to modulate numerous genes in terms of their transcript levels and the ratios of their splice variants [36]. Thus, smoking and drinking may alter the splicing of specific oncogenes and tumor suppressors, increasing the risk of tumorigenesis among subjects with the risk variant of rs2074733 in SF3A1.

Two other SNPs of SF3A1 (rs5994293 and rs9608886), which also locate to the region of 22q12.2, were significantly associated with PC in screening population. However, these associations were not replicated in validation population. The same phenomenon was observed for PRPF40B rs4073998 and SRSF1 rs8819, suggesting that these positive associations in screening population may have been due to chance. In the future, additional studies in diverse populations, including PC patients with different stages, should be conducted to examine the possible associations between PC and these SNPs.

## MATERIALS AND METHODS

### Study subjects

In this two-stage case-control study, screening population was conducted among 298 PC patients and 525 cancer-free controls from Central China. Patients were consecutively recruited from January 2008 to September 2012 at Tongji Hospital (Huazhong University of Science and Technology, Wuhan, China). The controls were cancer-free volunteers randomly selected from heath examination programs given during the same period, part of which also included in our previous case-control studies [37]. Then, 413 PC patients and 557 cancer-free controls from North China were genotyped for validation. All cases were enrolled from January 2008 to December 2012 at Peking Union Medical College Hospital. The controls were cancer-free individuals selected from a community cancer-screening program for early detection, offered in the same region during the same time. All the PC patients in the two stages were diagnosed with pancreatic ductal adenocarcinoma by histopathologic examination of biopsy or resected tissue specimens, diagnostic imaging studies (computed tomography scan, ultrasound, and endoscopic retrograde cholangiopancreatography or magnetic resonance cholangiopancreatography, or exploratory laparotomy.

All enrolled subjects were unrelated Han Chinese. The cases were primary incident pancreatic ductal adenocarcinoma patients. The blood was collected prior to radiotherapy/chemotherapy. The cancer-free controls were frequency-matched to the cases by age (±5 years) and gender. Prior to recruitment, written informed consent was obtained from each subject, and information on demographic characteristics (e.g., gender, age, smoking status, and drinking status) was collected in a face-to-face interview. Regular smoking is defined as at least one cigarette a day and for a year or more. Regular drinking is defined as drinking at least once a day for three consecutive months or more. This study was approved by the institutional review boards of Chinese Academy of Medical Sciences Cancer Institute and the Tongji Medical College of Huazhong University of Science and Technology. All the methods were performed in accordance with the approved guidelines and regulations.

### SNP selection and genotyping

To choose candidate SNPs from the selected genes (SRSF1, SRSF2, SF3A1, SF3B1, SF1 and PRPF40B), we integrated information from the Haploview 4.2 (http://www.broadinstitute.org/scientific-community/science/programs/medical-and-population-genetics/haploview/haploview) [38] and SNP info (http://snpinfo.niehs.nih.gov/snpinfo/snpfunc.htm) [39] databases.

First, SNP data for the candidate gene regions were downloaded from the Hapmap database for the Chinese Han Beijing population (Release 27 phase I+II+III; http://www.HapMap.org). The Haploview 4.2 software was then used to select tagSNPs based on the criteria of *r* ^2^ > 0.8 and a minor allele frequency (MAF) > 0.05. We selected a total of 82 tagSNPs covering the six candidate genes. Thereafter, we used the SNPinfo database to prioritize the 82 tagSNPs by predicting their potential functions, which included splicing regulation, stop codon, polyphen prediction, transcription factor-binding motif, micro RNA binding site, amino acid substitution, and other functional effects. Finally, we selected 17 SNPs for genotyping of the screening population (Supplementary Table S1) using the TaqMan Openarray assay system (Applied Biosystems, Foster City, CA, USA). Thereafter, the five most positive SNPs were genotyped using TaqMan Real-time Polymerase Chain Reaction (RT-PCR, Applied Biosystems, Foster City, CA, USA) in the validation population. To ensure the accuracy of genotyping, quality control was monitored by including 5% random duplicate samples; these duplicates yielded a concurrence rate of 100% (data not shown). We excluded SNPs that had genotype call rates of < 95% or showed deviation from HWE.

### Statistical analysis

For each tagSNP, we evaluated HWE in both control groups using a goodness-of-fit chi-square test. In both populations, *t*-test was used to compare the difference of age distribution between cases and controls. Pearson chi-square tests were used to detect differences between cases and controls in the distributions of gender, smoking status, drinking status and SNPs. Unconditional logistic regression was used to calculate the ORs and their 95% CIs for associations between genotypes and PC in both stages after adjustment for covariates (i.e., age, gender, drinking status, and smoking status). A two-tailed *P* < 0.05 was used as the criterion of statistical significance. False discovery rate (FDR) is the expected ratio of erroneous rejections of the null hypothesis to the total number of rejected hypothesis among all the genes or SNPs analyzed in this study. The Benjamini and Hochberg method was used to calculate FDR values using in SAS [40]. All P values were adjusted by FDR, and we adjusted the P values in the validation stage and combined stage together with the P values in the screening stage.

Before we combined the populations of the two stages, the Breslow-Day test [41] was used to assess the homogeneity of the ORs in the two populations. And we added the stage for adjustment in the combined analysis [42]. Multiplicative and additive interactions were tested to evaluate positive interactions between genes and environmental factors. Multiple logistic regression models were used to detect the potential multiplicative interactions, and the log likelihood ratio (LLR) was used to assess whether the model was significantly improved by adding an additional interaction term. SI, AP, and RERI were evaluated in additive interaction analyses. Lack of interaction is indicated by SI = 1, AP = 0 and RERI = 0[43]. All statistical analyses were conducted using the SAS9.2 software (SAS Institute, Cary, NC, USA). The linkage disequilibrium of the SNPs was estimated using the Haploview4.2 software. The additive interaction analysis was performed using the R3.0.0 software (http://www.r-project.org/).

## CONCLUSIONS

We herein report SF3A1 rs2074733 as a new susceptibility locus for PC that shows additive interactions with smoking and alcohol drinking. These findings support the potential importance of genetic variants of splicing factors in PC and could help increase the personalization of strategies to prevent PC. Although SNPs with rare polymorphisms may have been missed in this study, our results suggest that additional studies with larger sample sizes should use targeted SNP fine mapping to further identify true causal variants, especially in 22q12.2. Furthermore, additional functional studies are warranted to verify the biological mechanism(s) underlying the interactions between smoking, drinking and spliceosome gene polymorphisms.

## SUPPLEMENTARY FIGURE AND TABLES


