# Supplementary material for: SF3A1 and pancreatic cancer: new evidence for the association of the spliceosome and cancer
Source: Oncotarget. 2015 Oct 15;6(35):37750–7. doi: 10.18632/oncotarget.5647 (PMC4741962; doi:10.18632/oncotarget.5647)
Supplement: Supplementary file 1 [file oncotarget-06-37750-s001.pdf]

SUPPLEMENTARY FIGURE AND TABLES

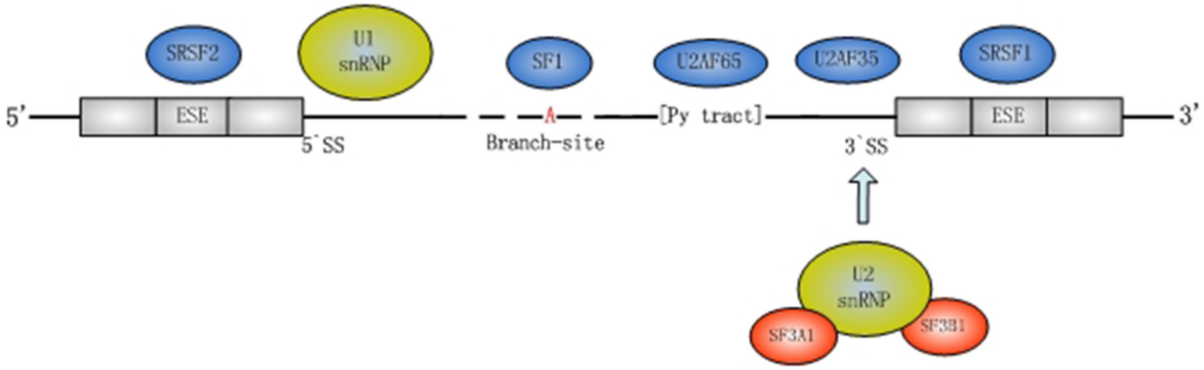

Supplementary Figure S1: The major components of the U2-dependent spliceosome. The U2-dependent intron recognition is initiated by the recruitment of U1 snRNP to the 5′SS. SF1 binds the branch-site A residue. The larger subunit of U2 snRNP auxiliary factor (U2AF),U2AF65, recognizes the downstream polypyrimidine tract (Py tract) of the branch site while the smaller subunit, U2AF35, binds to the AG dinucleotide of the 3′SS. U2 snRNP is recruited to the 3′SS with the help of SF3A1 and SF3B1. The SR proteins, such as SRSF1 and SRSF2, can bind to exonic splicing enhancers (ESEs) through the RS domain and help the recruiting of U1 snRNP and U2AF to the 5′ or 3′SS

Supplementary Table S1: Information on the 17 SNPs from six candidate spliceosome genes in the screening stage

Gene symbol	rsID	Position	Alleles	MAF	*P* for HWE	FDR-*P* for HWE[a]
PRPF40B	rs4073998	chr12 50038292	A/G	A	0.848	1
PRPF40B	rs8626	chr12 50034668	A/G	G	0.966	1
SF1	rs11231868	chr11 64543901	A/G	G	0	< 0.001
SF1	rs474707	chr11 64544135	C/T	T	0.848	1
SF3A1	rs12484880	chr22 30751453	G/T	G	1	1
SF3A1	rs2074733	chr22 30738587	C/T	T	0.698	1
SF3A1	rs5749066	chr22 30736911	A/G	G	0	< 0.001
SF3A1	rs5749068	chr22 30737015	C/T	T	0	< 0.001
SF3A1	rs5753081	chr22 30740653	C/T	T	0.771	1
SF3A1	rs5994293	chr22 30752696	G/T	G	0.131	0.445
SF3A1	rs7288947	chr22 30745221	C/T	T	0.574	1
SF3A1	rs8141656	chr22 30740148	C/T	C	0.995	1
SF3A1	rs9608886	chr22 30739394	G/T	G	0.541	1
SF3B1	rs11683572	chr2 198289575	C/G	G	0.087	0.370
SRSF1	rs2233911	chr17 56083934	A/G	G	0.942	1
SRSF1	rs8819	chr17 56078738	C/T	T	0.896	1
SRSF2	rs237059	chr17 74730843	C/T	T	0.99	1

[a]Each *P* value was modified by FDR correction for multiple comparisons (the number of comparisons = 17)

Supplementary Table S2: Effects of 14 tagSNPs from six RNA splicing-related genes on PC risk in the screening population

Gene	SNP	Genotypes[a]	Controls(*N* = 525) (%)	Cases(*N* = 298) (%)	OR(95%CI)[b]	*P*	FDR-*P*[c]
PRPF40B	rs4073998	(AA+AG)/GG	65(12.4)/460(87.6)	59(19.8)/239(80.2)	1.74(1.18–2.56)	0.005	0.014
	rs8626	(GG+AG)/AA	71(13.5)/454(86.5)	78(26.2)/220(73.8)	2.26(1.58–3.25)	8.98E-06	1.26E-04
SF1	rs474707	(TT+CT)/CC	320(61.0)/205(39.0)	178(59.7)/120(40.3)	0.97(0.73–1.31)	0.860	0.860
SF3A1	rs12484880	(GG+TG)/TT	76(14.5)/449(85.5)	47(15.8)/251(84.2)	1.11(0.75–1.66)	0.593	0.692
	rs2074733	(TT+CT)/CC	366(69.7)/159(30.3)	179(60.1)/119(39.9)	0.64(0.48–0.87)	0.004	0.014
	rs5753081	CT/CC	29(5.5)/496(94.5)	15(5.0)/283(95.0)	0.92(0.49–1.76)	0.808	0.860
	rs5994293	GG/GT/TT	56(10.7)/200(38.1)/269(51.2)	19(6.4)/102(34.2)/177(59.4)	0.75(0.60–0.94)	0.012	0.028
	rs7288947	(TT+CT)/CC	166(31.6)/359(68.4)	76(25.5)/222(74.5)	0.74(0.54–1.02)	0.067	0.134
	rs8141656	(CC+CT)/TT	261(49.7)/264(50.3)	164(55.0)/134(45.0)	1.24(0.93–1.65)	0.138	0.193
	rs9608886	(GG+GT)/TT	113(21.5)/412(78.5)	40(13.4)/258(86.6)	0.56(0.38–0.83)	0.004	0.014
SF3B1	rs11683572	(GG+CG)/CC	222(42.3)/303(57.7)	108(36.2)/190(63.8)	0.78(0.58–1.04)	0.089	0.145
SRSF1	rs2233911	(GG+AG)/AA	170(32.4)/355(67.6)	114(38.3)/184(61.7)	1.29(0.96–1.74)	0.093	0.145
	rs8819	CT/CC	21(4.0)/504(96.0)	32(10.7)/266(89.3)	3.08(1.73–5.49)	1.39E-04	0.001
SRSF2	rs237059	CT/CC	6(1.1)/519(98.9)	1(0.3)/297(99.7)	0.29(0.03–2.40)	0.249	0.317

[a]The last genotype was used as the reference for OR estimation.
[b]Adjusted by gender and age in the unconditional logistic regression.
[c]Each *P* value was modified by FDR correction for multiple comparisons.
